# Osthole enhances the immunosuppressive effects of bone marrow‐derived mesenchymal stem cells by promoting the Fas/FasL system

**DOI:** 10.1111/jcmm.16459

**Published:** 2021-03-21

**Authors:** Yang Yu, Meng Chen, Shiyao Yang, Bingyi Shao, Liang Chen, Lei Dou, Jing Gao, Deqin Yang

**Affiliations:** ^1^ Northern Department of Endodontics Stomatological Hospital of Chongqing Medical University Chongqing China; ^2^ Chongqing Key Laboratory of Oral Diseases and Biomedical Sciences Chongqing China; ^3^ Chongqing Municipal Key Laboratory of Oral Biomedical Engineering of Higher Education Chongqing China

**Keywords:** bone marrow‐derived mesenchymal stem cells, Fas/FasL, immunosuppressive therapy, osthole

## Abstract

Thanks to the advantages of easy harvesting and escape from immune rejection, autologous bone marrow‐derived mesenchymal stem cells (BMSCs) are promising candidates for immunosuppressive therapy against inflammation and autoimmune diseases. However, the therapy is still challenging because the immunomodulatory properties of BMSCs are always impaired by immunopathogenesis in patients. Because of its reliable and extensive biological activities, osthole has received increased clinical attention. In this study, we found that BMSCs derived from osteoporosis donors were ineffective in cell therapy for experimental inflammatory colitis and osteoporosis. In vivo and in vitro tests showed that because of the down‐regulation of Fas and FasL expression, the ability of osteoporotic BMSCs to induce T‐cell apoptosis decreased. Through the application of osthole, we successfully restored the immunosuppressive ability of osteoporotic BMSCs and improved their treatment efficacy in experimental inflammatory colitis and osteoporosis. In addition, we found the immunomodulatory properties of BMSCs were enhanced after osthole pre‐treatment. In this study, our data highlight a new approach of pharmacological modification (ie osthole) to improve the immune regulatory performance of BMSCs from a healthy or inflammatory microenvironment. The development of targeted strategies to enhance immunosuppressive therapy using BMSCs may be significantly improved by these findings.

## 1 INTRODUCTION

Autologous bone marrow‐derived mesenchymal stem cells (BMSCs) have strong immunosuppressive effects, and their application for the treatment of various autoimmune and inflammatory diseases, such as diabetes mellitus, lupus, Crohn's disease and osteoporosis,^1^, ^2^ has been evaluated worldwide.^3^ A reliable method to determine the immunomodulatory characteristics of BMSCs has become a key issue in BMSC cytotherapy. Emerging evidence suggests that BMSCs respond positively to the microenvironment because they are plastic stem cells, and the microenvironment can affect the immunosuppressive properties of exogenous BMSCs.^4^, ^5^

As shown in recent studies, BMSCs from different donors have different immunoregulatory characteristics. For instance, BMSCs isolated from healthy candidates are more effective than those derived from patients with lupus for the treatment of autoimmune diseases and the migration capacity of BMSCs harvested from patients with systemic lupus erythematosus is also impaired.^6^ In accordance with previous studies, we found that the treatment effect of BMSCs from osteoporosis mice on immune inflammatory diseases such as colitis is worse than that of BMSCs from normal mice. We also found that the immunoregulation of BMSCs was affected by the Fas / FasL pathway in osteoporosis. These findings suggest that the pathological condition of the donor may affect the immunosuppressive effects of BMSCs.^1^, ^7^ Although recent studies have shown that the recognition of small molecular compounds can counteract inflammatory insults in BMSCs,^8^ a pharmacological solution that promotes the immunosuppressive effects of BMSCs derived from inflammatory microenvironment has not yet been established.

Osthole is a natural pyroxanthin originally extracted from the Cnidium plant, which is commonly used in the clinical practice of traditional Chinese medicine.^9^ Osthole has been proven to have various functions, such as osteogenesis, hepatoprotection, cardiovascular protection, anti‐inflammation, anti‐tumour and anti‐microbial activities.^10^ Its immunoregulatory capability has been further demonstrated in bronchial epithelial cells, peripheral cone cells and peripheral blood mononuclear cells.^11^, ^12^ Osthole has been shown to induce apoptosis and cell cycle arrest in breast cancer cells by inhibiting signal transducer and activator of transcription 3 (STAT 3) phosphorylation and nuclear migration.^13^ However, the effects of osthole on the immunomodulatory properties of BMSCs derived from inflammatory and healthy microenvironments have not yet been evaluated. As an extension of our previous investigations,^1^, ^7^ this study attempts to assess the immunomodulatory ability potential of BMSCs from osteoporosis mice for the treatment of colitis and osteoporosis. In the present study, we used osthole‐modified BMSCs to improve the immunoregulatory function in vitro, to treat inflammatory colitis and oestrogen deficiency‐induced osteoporosis in a mice model, and to investigate the underlying mechanisms.

## 2 MATERIALS AND METHODS

### 2.1 Animals

All animal experiments were conducted under the experimental animal guidelines of the National Institutes of Health and approved by the Animal Care and Use Committee of Chongqing Medical University. Female C57BL/6 mice (21‐25 g) aged 8 weeks were purchased from the animal centre of Chongqing Medical University. All mice were bred in specific pathogen‐free conditions and had free access to food and water. C57BL/6 mice were used as the sources for the osteoporosis model, experimental colitis model, BMSC culture and other cell experiments. Dextran sulphate sodium salt (DSS; 3%; MP Biomedical, Irving, California, USA) was fed to the mice in water for 10 days to induce experimental colitis. To establish the oestrogen deficiency‐induced osteoporosis model, the mice were anaesthetized and received bilateral ovariectomy or sham operation.

### 2.2 BMSC and T‐cell isolation and coculture

BMSCs isolated from the femurs and tibias and T cells isolated from the spleen of C57BL/6 mouse were cultured as described previously.^1^ BMSCs were passaged for three generations, and the purity and specificity were verified by conventional characterization, surface marker identification, and self‐renewal and multi energy differentiation assays. BMSCs from healthy sham and osteoporosis donors were transplanted and designated as S/BMSCs and O/BMSCs, respectively.

The spleen single cell suspension was harvested by crushing the spleen of the mice. After red blood cells were removed using a red blood cell lysis buffer (Sigma‐Aldrich, St. Louis, USA), spleen cells were cultured with RPMI 1640, supplemented with 10% foetal bovine serum, 50 mm 2‐ME, 2 mm L‐glutamine (Sigma‐Aldrich) and 1% penicillin streptomycin. Anti‐CD3 and anti‐CD28 (1 mg/mL; Santa Cruz Biotechnology) were used to activate T‐cell proliferation for 48 hours. A total of 2 × 10^6^ activated T cells were then added to six‐well plates pre‐seeded with 2 × 10^5^ BMSCs for 48 hours of coculture and further analysis.

### 2.3 BMSC pre‐treatment with osthole

Osthole with purity above 98% was purchased from Medchem Express (Shanghai, China). In accordance with previous studies,^14^ confluent BMSCs were incubated with basal medium containing 10^‐5^ mol/L osthole for 3 days and the resulting osthole‐modified BMSCs were used for subsequent studies.

### 2.4 BMSC cytotherapy in animal model

For the experimental colitis model, 25 8‐week‐old C57BL/6 mice were randomly and equally distributed into five groups (H_2_O, DSS, O/BMSCs, S/BMSCs and O/BMSCs + Osthole). On day 3 after the experimental colitis model was established, 1 × 10^6^ osthole‐modified BMSCs were injected into the mice via the tail vein. Bodyweight, survival rate and stool were recorded daily. At the end of the experiment, the colons of the mice were collected for histopathological analysis. The disease index and histologic score were assessed in accordance with previously published criteria.^15^ To explore the effects of a systemic infusion of BMSCs for oestrogen deficiency‐induced osteoporosis, 25 8‐week‐old C57BL/6 mice were randomly and equally distributed into five groups (Sham, OVX, O/BMSCs, S/BMSCs and O/BMSCs + Osthole). To investigate the impact of osthole‐modified BMSCs on osteoporosis, 20 8‐week‐old C57BL/6 mice were randomly and equally distributed into four groups (Sham, OVX, S/BMSCs and S/BMSCs + Osthole). The mice received 1 × 10^6^ osthole‐modified BMSCs transplantation on day 7 (mice aged 9 weeks) after ovariectomy and were killed on day 28 when the mice were aged 13 weeks.^16^, ^17^

### 2.5 T‐cell counting, apoptosis assay and migration assay

The spleens of mice fed DSS for 10 days were isolated and crushed to obtain a single cell suspension. After red blood cell lysis, the cell suspension was washed twice with phosphate‐buffered saline (PBS) and incubated with fluorescent CD3 Ab (1:1000; BD Pharmingen) for 1 hour. In accordance with a previous study,^1^ CD3 + T cells in the cell suspension were counted using flow cytometry (BD Bioscience).

To explore the apoptosis of T cells in the osteoporosis model, the spleen cell suspension was stained with CD3 antibody (1:1000) (Santa Cruz Biotechnology) for 30 minutes, and apoptosis analysis of the T cells was performed with an Annexin V Apoptosis Detection Kit I (BD Bioscience) following the instructions. Flow cytometry (BD Bioscience) was performed to analyse apoptosis in CD3 + T cells.

T‐cell migration assays were performed in accordance with previous reports.^1^ Briefly, in a Transwell system (Corning), 2 × 10^5^ BMSCs labelled with a PKH26 Red Fluorescent Kit (5 mmol/L; Sigma‐Aldrich) were seeded in the lower chamber for 24 hours, while 1 × 10^5^ activated CD3 + T cells were stained with 5 mg/mL of Hoechst‐33342 (Sigma‐Aldrich) for 30 minutes and seeded in the upper chamber. After 48 hours of coculture, the T cells that migrated to the lower chamber were counted under a fluorescence microscope (Olympus Optical, Tokyo, Japan).

### 2.6 Gene expression assay

The level of monocyte chemotactic protein 1 (MCP‐1) in the supernatants of BMSCs and tumour necrosis factor (TNF)‐α and interferon (IFN)‐γ levels in the serum were measured using commercialized inflammatory cytokine ELISA kits (Beyotime, Haimen, China). For Western blot analysis, primary antibodies (Santa Cruz Biotechnology) were used to detect the protein expression of Fas, FasL and β‐actin. Enhanced chemiluminescence (ECL) kits (Amersham Biosciences) were used to explore the blots on the membranes. ImageJ software was used to quantify the grey values of blots as described previously.^18^ Real‐time reverse transcription polymerase chain reaction (RT‐PCR) analysis using the primers listed in Table 1 was performed as previously described to measure the mRNA expression of Fas, FasL and β‐actin.^18^

**TABLE 1:** RT‐PCR primers

Genes	Primer sequences
β‐actin	Forward: 5’‐CTGGCACCACACCTTCTACA‐3’
	Reverse: 5’‐GGTACGACCAGAGGCATACA‐3’
FasL	Forward: 5’‐CTGGGTTGTACT TCGTGTATTCC‐3’
	Reverse: 5’‐TGTCCAGTAGTGCAGTAGTTCAA‐3’
Fas	Forward: 5’‐TGCATGACAGCATCCAAGACA‐3’
	Reverse:5’‐GCACAGGAGCAGCTGGACTT‐3’

### 2.7 Micro‐computed tomography (CT) examination

Micro‐CT (GE, Germany) was used to scan the femurs. The X‐ray source was set to 80 kV and 500 μ for a micro focus. Areas of interest were selected manually in the marrow cavity. Bone morphology parameters were evaluated, including bone mineral density (BMD), bone volume/tissue volume (BV/TV), trabecula number (Tb.N), trabecula thickness (Tb. Th) and trabecular thickness (Tb. Sp).

### 2.8 Statistical analysis

The data were presented as the mean ± SD. Comparisons were conducted using Student's t test or one‐way ANOVA. Survival was compared with the Kaplan‐Meier log‐rank test, whereas bodyweight changes were analysed with the matched‐pair signed‐rank test. Each experiment was performed with at least three replicates. Differences were considered significant at *P* values < .05.

## 3 RESULTS

### 3.1 Osthole restores the O/BMSC‐mediated T‐cell migration and apoptosis in vitro

**FIGURE 1:**
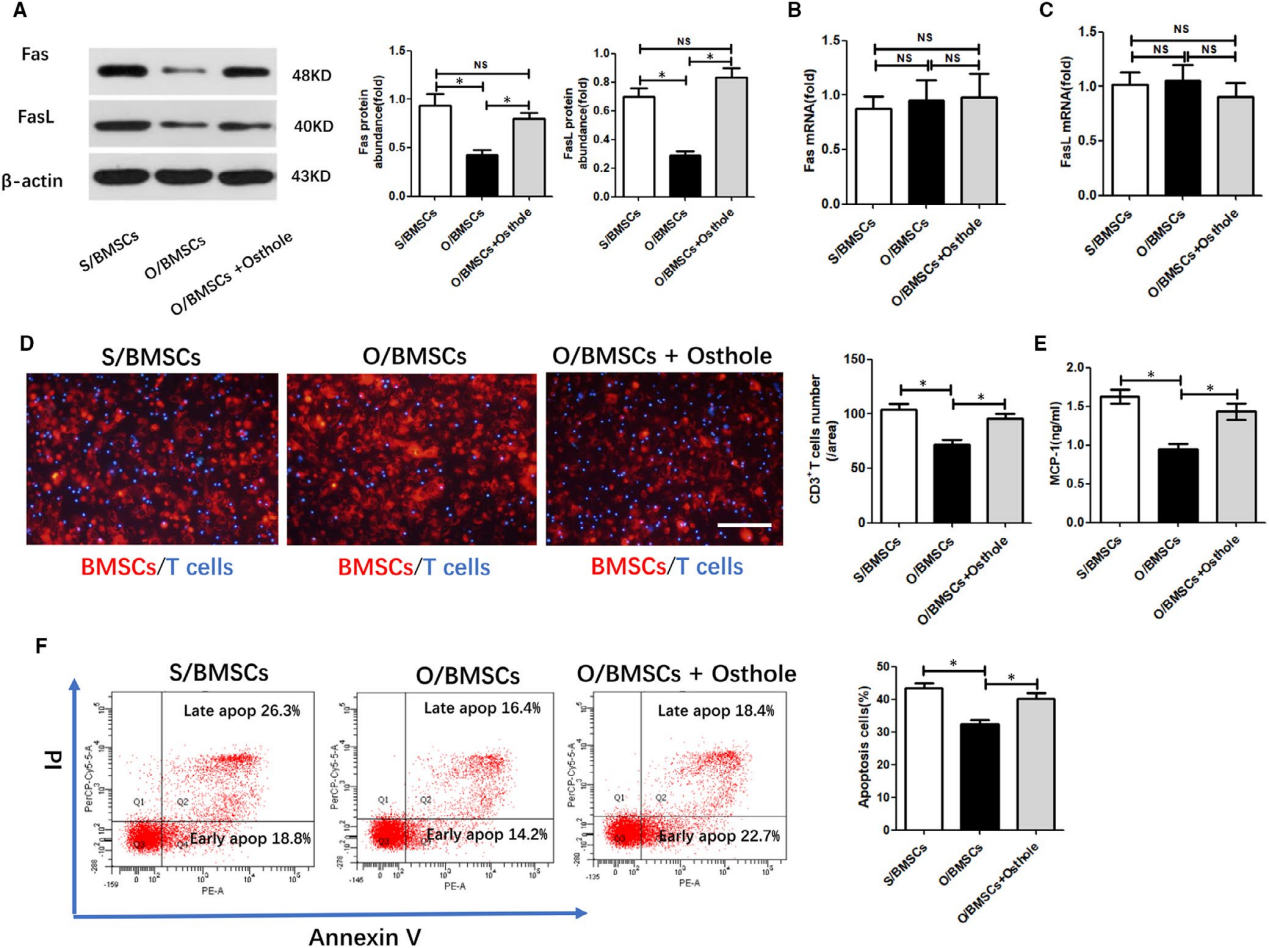
Osteoporotic BMSC‐induced T‐cell migration and apoptosis were rescued by osthole. (A) Western blot of Fas and FasL protein accumulation in BMSCs. ImageJ was used to measure the relative protein abundance. (B, C) The mRNA levels of Fas and FasL were measured with real‐time PCR. (D) T cells were cocultured with S/BMSCs, O/BMSCs and O/BMSCs pre‐treated with osthole. A fluorescence microscope was used to quantify and observe T‐cell migration. Scale bar, 100 mm. (E) MCP‐1 levels in the culture medium of S/BMSCs, O/BMSCs and O/BMSCs pre‐treated with osthole were measured with ELISA. (F) Flow cytometry was used to analyse apoptotic T cells induced by S/BMSCs, O/BMSCs and O/BMSCs pre‐treated with osthole. NS, not significant; data presented as mean = ± SD. *P <.05 and **P <.01

The BMSCs used in our study highly expressed mesenchymal stem cell markers (Sca‐1 and CD29) were negative for haematopoietic cell markers (CD34, CD45 and CD146) (Figure S1A) and possessed self‐renewal abilities (Figure S1B) and multi‐potent differentiation properties (Figure S1C,D). Our previous study demonstrated that because Fas and FasL accumulation in O/BMSCs was reduced, the capacity of BMSCs to induce T‐cell migration and apoptosis was impaired in oestrogen deficiency‐induced osteoporosis compared with that of S/BMSCs. We further investigated whether osthole can restore O/BMSC‐mediated T‐cell migration and apoptosis via stimulating the expression of Fas and FasL. It is worth noting that Western blot showed that osthole pre‐treatment increased the accumulation of Fas and FasL proteins in O/BMSCs (Figure 1A), although osthole pre‐treatment did not induce significant changes in the levels of Fas and FasL mRNA (Figure 1B,C). Because only FasL bound to the BMSC membrane can mediate Fas‐induced apoptosis in adjacent T cells, BMSCs must recruit T cells first.^19^ Using the Transwell system, we confirmed that osthole pre‐treatment did promote T‐cell migration triggered by O/BMSCs (Figure 1D). As an explanation for the T‐cell migration modulation, we found that pre‐treatment with osthole significantly increased MCP‐1 secretion by O/BMSCs (Figure 1E), which is consistent with the early findings indicating that MCP‐1 is a key chemokine secreted by BMSCs to promote T‐cell migration.^1^ Next, we tested whether osthole pre‐treatment could promote O/BMSC‐induced apoptosis of T cells using a direct coculture model in vitro. The apoptosis assay showed that compared with S/BMSCs, osthole‐modified O/BMSCs increased the apoptosis rate of T cells, while naïve O/BMSCs reduced the apoptosis rate (Figure 1F). These findings illustrated that osthole can rescue O/BMSC‐induced T‐cell migration and apoptosis.

### 3.2 Osthole rescues the O/BMSC‐mediated immunosuppression in colitis model

**FIGURE 2:**
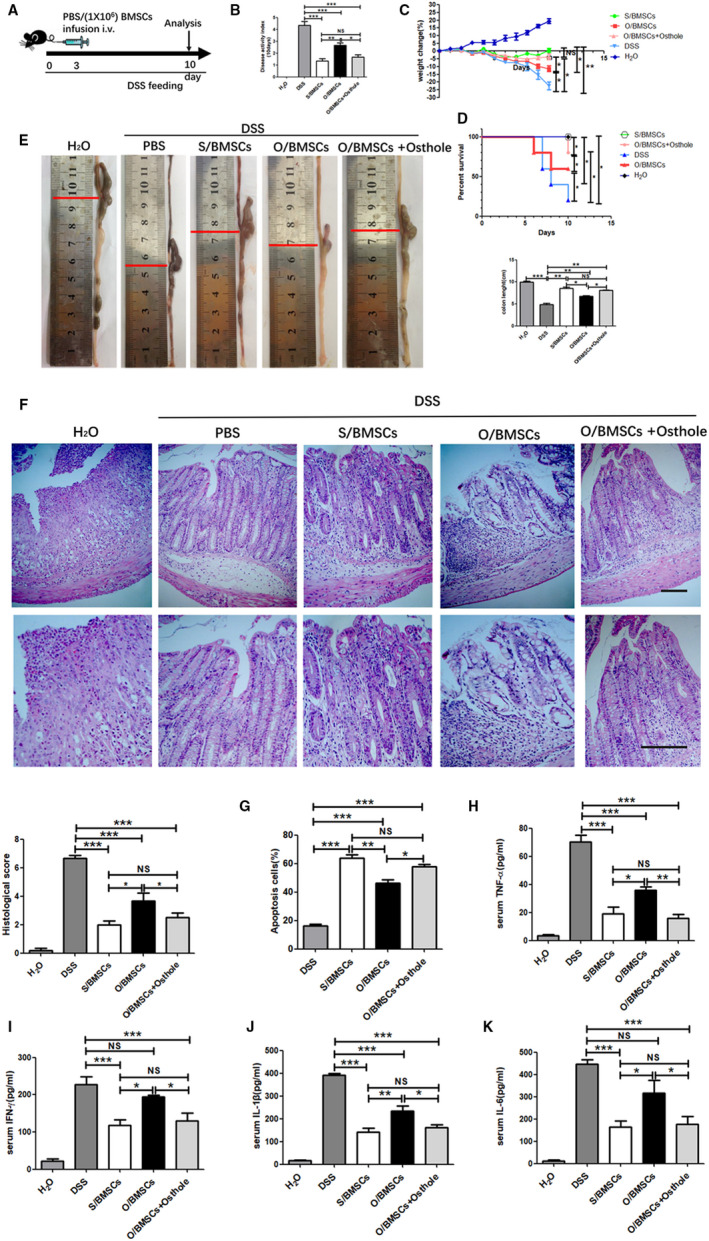
Treatment of inflammatory colitis with BMSCs. (A) Experimental design. Mice were fed drinking water containing DSS for 10 days. On the third day, 1 × 10^6^ S/BMSCs, O/BMSC and O/BMSCs pre‐treated with osthole were injected into the mice through the tail vein. (B) The disease index was measured on the 10th day of DSS feeding. (C) Bodyweight was recorded every day for 10 days. (D) The mortality of the mice was recorded at 10 days. (E) The colon was collected 10 days later for each group, and the length was measured. (F) The histological structure of the colon was determined using H & E staining, and the histological score was graded. The bottom image is a higher magnification of the top image. Scale bar, 200 mm. (G) Ratio of apoptotic CD3 + T cells. (H, I, J, K) ELISA analysis of serum levels of inflammation markers TNF‐α, IFN‐γ, IL‐1β and IL‐6. NS, not significant; data displayed as means ± SD. N = 5 / group. *P <.05 and **P <.01

We further explored whether osthole pre‐treatment can restore the therapeutic effect of O/BMSCs in vivo (Figure 2A). Ten days after oral administration of DSS, significant weight loss, diarrhoea, faecal bleeding and mortality were observed in PBS‐injected mice. As expected, O/BMSCs pre‐treated with osthole were more effective than naïve O/BMSCs in preventing weight loss, reducing mortality and alleviating colitis symptoms (Figure 2B‐D). We next harvested colon specimens for histological examination. BMSC injections improved the colon length reduction (Figure 2E), lymphocyte infiltration and mucosal damage caused by DSS. Consistent with the improvement of symptoms, O/BMSCs pre‐treated with osthole improved lymphocyte infiltration and tissue damage more effectively than naïve O/BMSCs (Figure 2F). Flow cytometry results showed that mice with colitis infusion of O/BMSCs had a relatively low cell apoptosis rate, but O/BMSCs pre‐treated with osthole could significantly elevate the CD3 + T‐cell apoptotic rate to a level comparable to that of S/BMSCs (Figure 2G). ELISA results showed that S/BMSCs, together with osthole‐modified O/BMSCs, significantly inhibited TNF‐α, IFN‐γ, interleukin (IL)‐1β and IL‐6 levels in the serum (Figure 2H‐K). Taken together, these results showed that osthole pre‐treatment rescues the immunosuppressive capacity of O/BMSCs in vivo.

### 3.3 Osthole rescues the O/BMSC‐mediated immunosuppression in osteoporosis model

**FIGURE 3:**
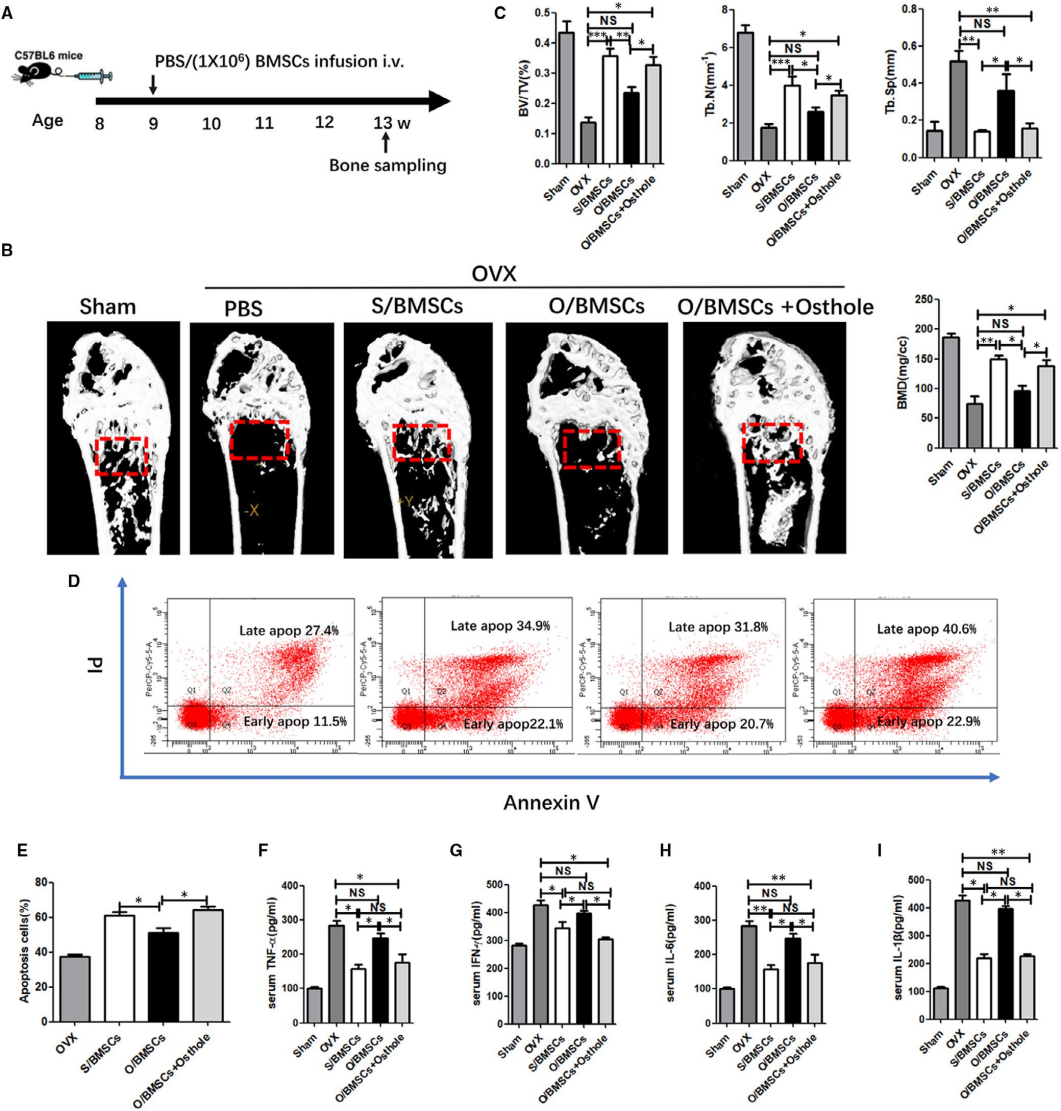
Effects of systemic infusion of S/BMSCs, O/BMSCs or O/BMSCs pre‐treated with osthole in osteoporosis mice. (A) Research design for bone mass evaluation. Femur and tibia were harvested at the time of animal sacrifice. (B) Representative micro‐CT images of the total bone mass of the femur harvested on day 28 post‐operation. (C) BMD, BV/TV, Tb.N and Tb. Sp were analysed with micro‐CT (n = 5/group). (D, E) Flow cytometry analysis of apoptotic CD3 + T cells. (F, G, H, I) ELISA analysis of the serum levels of inflammation markers TNF‐α, IFN‐γ, IL‐1β and IL‐6. NS, not significant; data are shown as means ± SD. N = 5 / group. *P <.05, **P <.01 and ***P <.001. Not significant (*P* >.05)

It has been reported that the ovariectomy model simulates bone pathogenesis caused by microenvironmental changes in oestrogen deficiency and secondary inflammation. Therefore, we next investigated whether our method was also suitable for the treatment of osteoporosis induced by ovariectomy through intravenous injections of O/BMSC, S/BMSCs and osthole‐modified O/BMSCs in ovariectomy‐induced osteoporosis mice (Figure 3A). As shown in Figure 3B and C, the micro‐CT images and corresponding bone parameters showed that O/BMSCs failed to protect the bone, but both S/BMSCs and osthole‐modified O/BMSCs effectively prevented cortical bone damage and bone microstructure damage. Flow cytometry assay showed that osteoporosis mice administrated with naïve O/BMSCs had a relatively low cell apoptosis rate. In contrast, O/BMSCs pre‐treated with osthole significantly elevated the CD3 + T‐cell apoptotic rate to a level comparable to that of S/BMSCs (Figure 3D‐E). We and others have found that elevated inflammatory cytokines, such as TNF‐α, IFN‐γ and IL‐1β, contribute to the pathogenesis of bone damage.^14^, ^18^ We found that S/BMSCs, together with O/BMSCs pre‐treated with osthole, significantly inhibited TNF‐α, IFN‐γ, IL‐1β and IL‐6 (Figure 3F‐I) levels compared to naïve O/BMSCs. These results collectively indicated that osthole can rescue the immunosuppressive effects of O/BMSCs against experimental osteoporosis in vivo.

### 3.4 Osthole increases S/BMSC‐induced T‐cell migration and apoptosis

**FIGURE 4:**
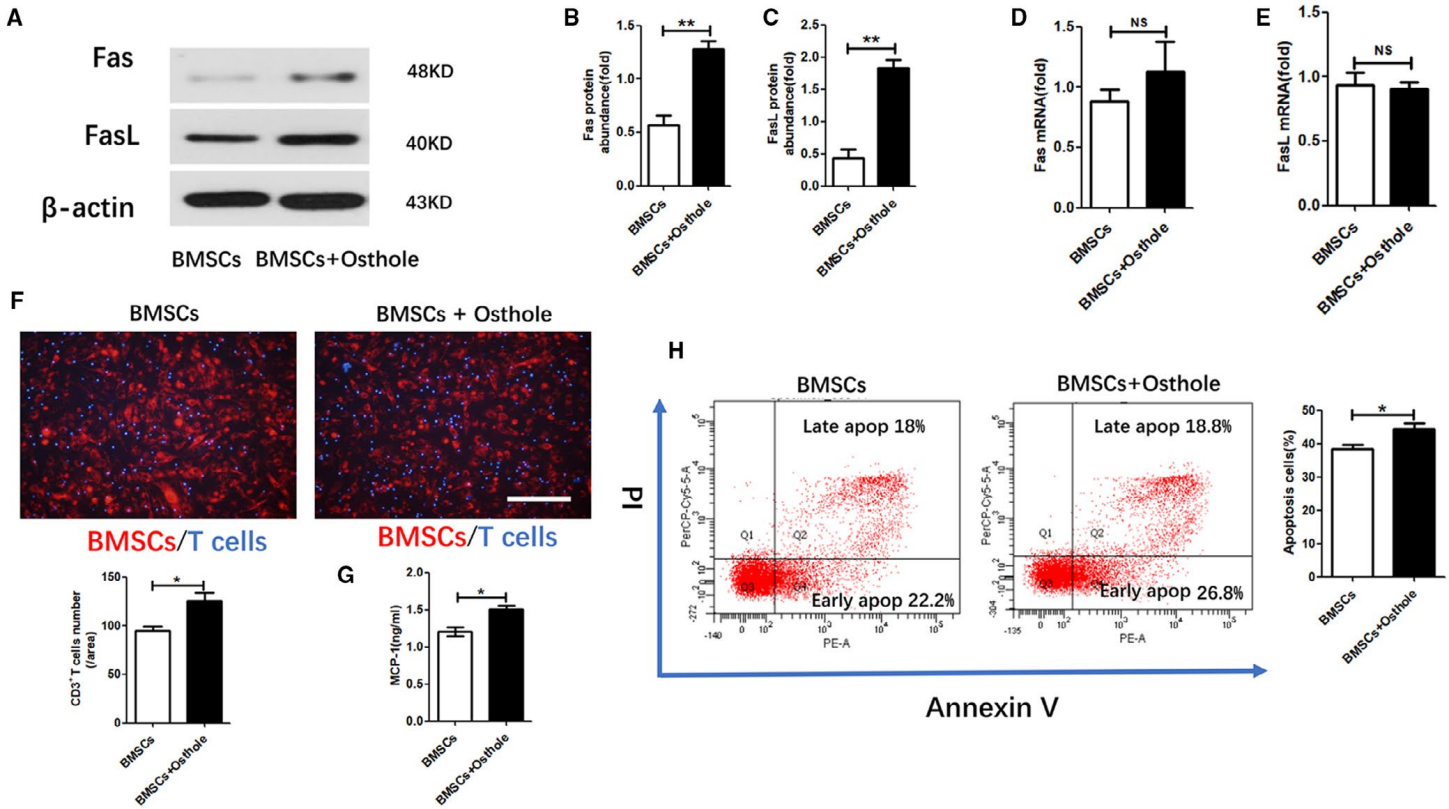
Osthole increased BMSC‐induced T‐cell migration and apoptosis. (A, B, C) Western blot was used to visualize the accumulation of Fas and FasL proteins in BMSCs. ImageJ software was used to measure the relative protein abundance. The grey value of each stain was normalized to the value of β‐actin. (D, E) Real‐time PCR was used to measure the mRNA levels of Fas and FasL. (F) T cells were cocultured with BMSCs pre‐treated with osthole. Under a fluorescence microscope, T‐cell migration was counted and quantified. Scale bar, 100 mm. (G) MCP‐1 level in BMSC culture medium was measured with ELISA. (H) Flow cytometry analysis of apoptotic T cells. NS, not significant; data are shown as means ± SD. *P <.05 and **P <.01

We also investigated whether osthole pre‐treatment can improve the expression of Fas and FasL in BMSCs from healthy donors. Western blotting showed that osthole pre‐treatment increased the accumulation of Fas and FasL proteins in **S/**BMSCs (Figure 4A‐C). However, after pre‐treatment, we did not observe substantial changes in Fas and FasL at the mRNA level (Figure 4D‐E). We confirmed that **S/**BMSCs pre‐treated with osthole can promote T‐cell migration compared to the control (Figure 4F). We also found that pre‐treatment with osthole increased the amount of S/BMSC‐secreted MCP‐1, a key chemokine for T‐cell recruitment (Figure 4G). Next, by using the direct coculture model in vitro, we explored whether BMSCs pre‐treated with osthole could promote the apoptosis of adjacent T cells. Apoptosis assays showed that compared to naïve S/BMSCs, osthole‐modified S/BMSCs increased the apoptosis rate of cocultured T cells (Figure 4H). These findings indicated that the immunomodulatory effects of BMSCs from healthy individuals can also be improved by osthole pre‐conditioning through increased T‐cell migration and apoptosis in vitro.

### 3.5 Osthole enhanced S/BMSC cytotherapy effects in osteoporosis model

**FIGURE 5:**
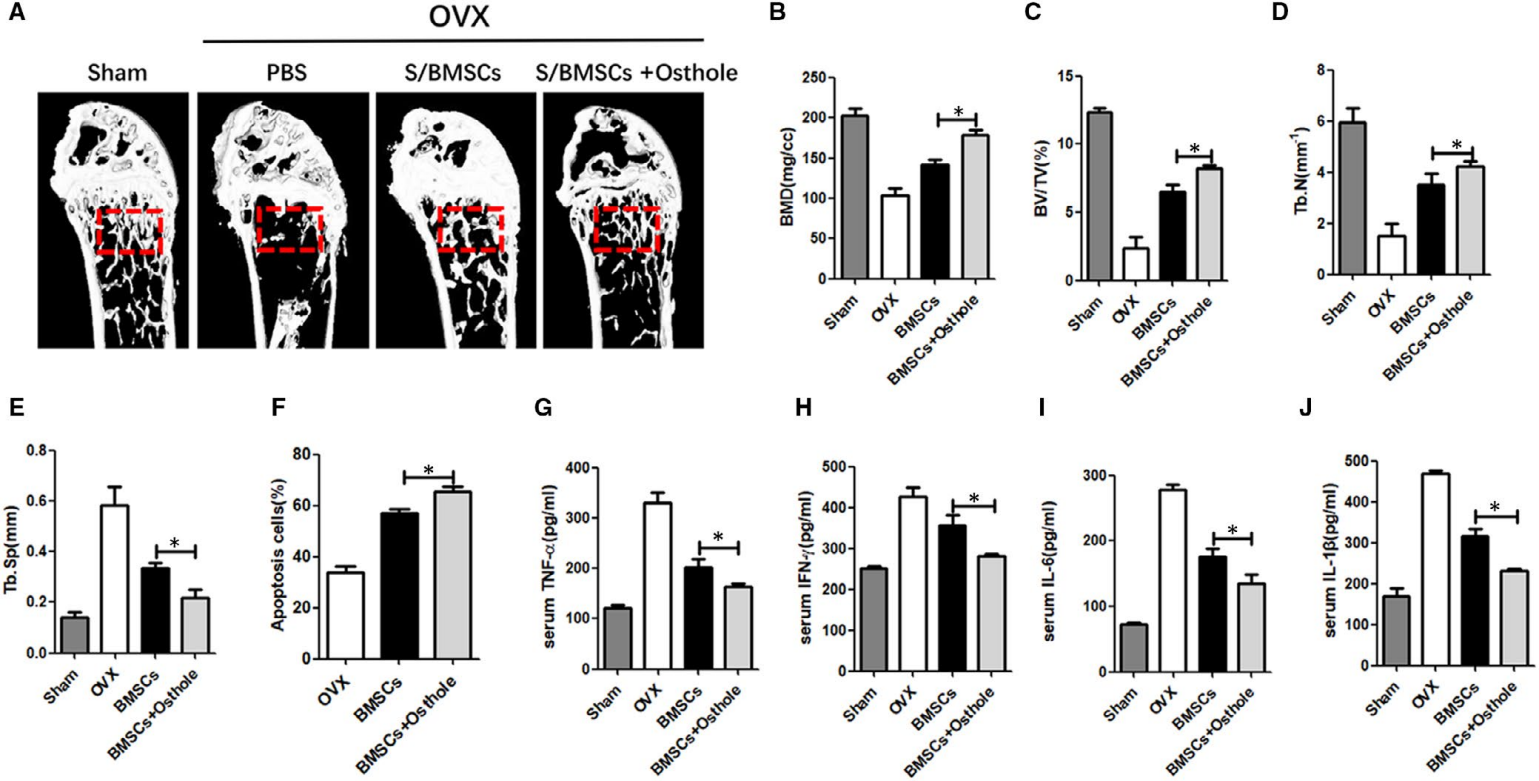
Osthole improved BMSC‐mediated immunotherapy for osteoporosis. (A) Typical micro‐CT images of the total bone mass of the femur were obtained at 28 days after surgery. BMD (B) BV/TV (C) Tb.N (D) and Tb. Sp (E) were analysed with micro‐CT. (F) Ratio of apoptotic CD3 + T cells. ELISA analysis of the serum levels of inflammation markers TNF‐α, IFN‐γ, IL‐1β and IL‐6. (G, H, I, J). Data are shown as means ± SD. N = 5 / group.*P <.05, **P <.01 and ***P <.001. NS, not significant (*P* >.05)

Next, we injected naïve S/BMSCs and osthole‐modified S/BMSCs into ovariectomy‐induced osteoporosis recipients. The data showed that compared with naïve S/BMSCs, the systemic infusion of S/BMSCs pre‐treated with osthole was more effective at preventing bone microstructure damage and cortical bone loss, as shown in the representative micro‐CT image (Figure 5A) and corresponding bone parameters (Figure 5B‐E). Flow cytometry results showed that mice with osteoporosis infused with naïve S/BMSCs had a relatively low T‐cell apoptosis rate. After treatment with S/BMSCs pre‐conditioned with osthole, the ratio of apoptotic CD3^+^ T cells increased (Figure 5F) and the inhibition of TNF‐α, IFN‐γ, IL‐1β and IL‐6 (Figure 5G‐J) was more significant. These results demonstrated that osthole can improve the therapeutic effects of osthole‐modified S/BMSCs against osteoporosis.

## 4 DISCUSSION

Cytotherapy based on BMSCs is a promising strategy for the treatment of autoimmune and inflammatory diseases,^1^, ^2^ but the impaired immune modulation properties of BMSCs derived from a diseased environment, especially an inflammatory condition, is the main obstacle to the clinical efficacy of BMSCs. New evidence suggests that physiological and pathological conditions affect the immunomodulatory properties of BMSCs. Therefore, it is imperative to improve the therapeutic effects of BMSCs in both physiological and pathological conditions. Recently, FasL/Fas was found to be important for the induction of T‐cell apoptosis and inhibition of the immune response by BMSCs.^1^, ^7^, ^15^ Here, we demonstrated that the pathological microenvironment of osteoporosis results in a defect in the immunosuppressive capacity of BMSCs, but this functional defect can be recovered by the osthole‐induced up‐regulation of Fas/FasL expression. We also found that the immunomodulatory ability of BMSCs from healthy individuals can be improved by osthole.

A growing number of studies have confirmed that inflammation plays an important role in osteoporosis and colitis.^1^, ^2^, ^14^ Pro‐inflammatory cytokines are important risk factors in the pathogenesis of osteoporosis.^14^ In experimental colitis, activated T cells facilitate the infiltration and activation of macrophages, leading to the production of numerous inflammatory cytokines and chemokines.^15^ Therefore, one of the most common treatments for both osteoporosis and colitis involves reducing pro‐inflammatory cytokine production through immunosuppression,^14^, ^15^ which makes these two diseases a promising model for BMSC‐mediated cytotherapy. Inflammatory diseases caused by cell dysfunction include inflammatory colitis, osteoporosis and graft versus host disease (GVHD).^1^, ^15^ At present, there is no ideal treatment with an ideal therapeutic rate for these diseases. We and others have reported that the key driving forces of pro‐inflammatory cytokines, such as TNF‐α, IL‐1β and IFN‐γ, accumulate in inflammatory colitis.^2^, ^5^ We also demonstrated that the BMSCs derived from healthy donors induced T‐cell apoptosis and further reduced pro‐inflammatory cytokine expression after systematic injection into mice.^20^ However, the reasons for the main differences in the anti‐inflammatory performance of donor‐derived BMSCs under different conditions are not fully understood. Some studies have shown that the therapeutic effects of autologous BMSC treatment for patients with osteoporosis are not ideal. Systematic infusion of allogeneic BMSCs was found to diminish Th1 and Th17 responses while increasing Th2 responses and T‐cell apoptosis in multiple sclerosis models.^21^, ^22^ However, Zhou et al reported a higher Th1/Th2 ratio in four allogeneic BMSC‐treated patients with sclerodermatous chronic GVHD, suggesting the allogeneic BMSC‐mediated inflammatory suppression needs to be enhanced.^23^ Several methods have been developed to improve the immunosuppressive capacity of allogeneic BMSCs, including microRNA transfection and Treg co‐transfusion.^7^, ^20^ However, autologous BMSCs have a numerical advantage and cannot be replaced by allogenic BMSCs in clinical practice.^24^ Therefore, approaches to improve the immunoregulatory properties of osteoporotic BMSCs are valuable. Recently, several genetic methods were used to improve BMSC therapy.^25^, ^26^ For example, BMSCs transduced with transforming growth factor (TGF)‐β using gland expression vectors are more effective at improving experimental autoimmune arthritis.^27^ However, despite the discovery of various molecular mediators affected by inflammation in BMSCs, pharmacological solutions to promote the immune regulation of BMSCs produced in inflammatory conditions remain largely unavailable. A few studies have reported the anti‐inflammatory effects of osthole on BMSCs. Previous studies found that osthole can significantly reduce the expression of Bcl‐2 and increase the expression levels of Bcl‐2‐associated X protein (Bax), Bcl‐2 antagonist killer 1 protein (BAK), Bcl‐2‐interacting mediator of cell death (BIML), and tBid as well as death receptor ligands and other components of the death receptor signalling pathway such as Fas, Fas‐associated protein with death domain (FADD), TNF receptor 1 (TNF‐R1), TNF‐R2, decoy receptor 2 (DcR2), receptor‐interacting protein (RIP) and death receptor 5 (DR5). Osthole can exert cytotoxic effects on nasopharyngeal carcinoma cell lines through apoptosis jointly mediated by Fas‐FasL and the mitochondrial pathway.^28^ ^,^ ^29^ In this study, we developed a new approach to improve the therapeutic activity of BMSCs through pharmaceutical modification with osthole. We found that osthole significantly improved BMSCs in the treatment of inflammatory colitis and osteoporosis caused by oestrogen deficiency. Considering that T cells play an important role in the development of inflammatory colitis and osteoporosis caused by oestrogen deficiency, and an abnormal and excessive activation of T cells can exacerbate colitis and osteoporosis,^31^, ^32^, ^33^ our results indicate that osthole pre‐treatment of BMSCs may be an effective method for the treatment of T cell‐induced inflammatory diseases. In view of the ability to improve cellular immunoregulation with osthole,^34^, ^35^ it is important to determine whether osthole can improve the effect of BMSC treatment in other inflammatory diseases.

In addition, according to previous research, moderate enhancement of FasL/Fas expression is a promising strategy to improve BMSC cytotherapy at the molecular level.^1^, ^30^ To establish immune tolerance, Fas can reduce T‐cell recruitment by decreasing the secretion of the chemokine MCP‐1 in BMSCs, while FasL expressed by BMSCs can induce T‐cell apoptosis in vitro and in vivo.^15^ We found that osthole can enhance the level of FasL/Fas protein by stimulating the Fas/FasL pathway, resulting in the promotion of Fas‐induced T‐cell migration and Fas‐induced T‐cell apoptosis. Recently, Zhu J and colleagues confirmed that the Fas/FasL system is important for T‐cell apoptosis in inflammatory and immune diseases.^36^ Not surprisingly, we also found that pre‐treatment with osthole‐modified BMSCs from healthy donors and significantly improved the therapeutic effects in inflammatory colitis and osteoporosis. To our knowledge, this is the first study to report that osthole can increase the immunomodulatory capacity of both osteoporotic BMSCs and healthy BMSCs. The results showed that stimulating Fas/FasL with osthole leads to the activation of T‐cell migration and apoptosis and alleviates osteoporosis and inflammatory colitis. Interestingly, we also found that osthole up‐regulated Fas and FasL at the protein level but not at the mRNA level. As important modulators of cell function, microRNAs (miRNAs) can inhibit gene expression post‐transcriptionally by binding to the 3’UTR of mRNA.^1^ We speculated that the reason osthole up‐regulated Fas and FasL at the protein but not mRNA level may be due to the fact that osthole inhibits the expression of some miRNAs.^37^, ^38^, ^39^ It is also important to explore whether existing miRNAs can be regulated by osthole.

Previous studies have shown that BMSCs genetically modified with viral vectors harbour potential risks in clinical applications ^40^; therefore, natural small molecules would be more practical and safer for the improvement of cell therapy. Our findings, combined with other previously published results,^41^, ^42^ indicate that osthole‐based cell therapy is an ideal optional solution to improve cytotherapy mediated by BMSCs derived from either healthy or inflammatory conditions. Our results further demonstrate that the use of allogeneic BMSCs without gene manipulation for the clinical treatment of immune and inflammatory diseases is feasible.

In conclusion, our data revealed a new approach of pharmaceutical modification as a functional and convenient solution to improve the immune regulation of BMSCs derived from healthy and inflammatory conditions, and provide a method to improve the clinical application of autologous BMSC‐based immunotherapy.

## CONFLICT OF INTEREST

The authors declare no competing financial interests.

## AUTHOR CONTRIBUTION

**Yang Yu:** Conceptualization (equal); Data curation (equal); Funding acquisition (equal); Investigation (equal); Writing–original draft (equal); Writing–review & editing (equal). **Meng Chen:** Investigation (equal). **Shiyao Yang:** Data curation (equal). **Bingyi Shao:** Investigation (equal). **Liang Chen:** Data curation (equal). **Lei Dou:** Investigation (equal). **jing Gao:** Investigation (equal). **Deqin Yang:** Funding acquisition (equal); Supervision (equal); Writing–review & editing (equal).

## Supporting information

Fig S1

## Data Availability

Data available on request from the authors.
