# Corrigendum

**DOI:** 10.1111/jcmm.17567

**Published:** 2022-12-15

Additional article information.

In Yang Yu et al.,^1^ the published article contains several image errors. Western Blot in Figure 1A, and Figure 4A–C, H & E staining in Figure 2F, flow cytometry in Figure S1A and coloning forming in Figure S1B are incorrect. The correct figures are shown below. We confirm all results and conclusions of this article remain unchanged.

**FIGURE 1:**
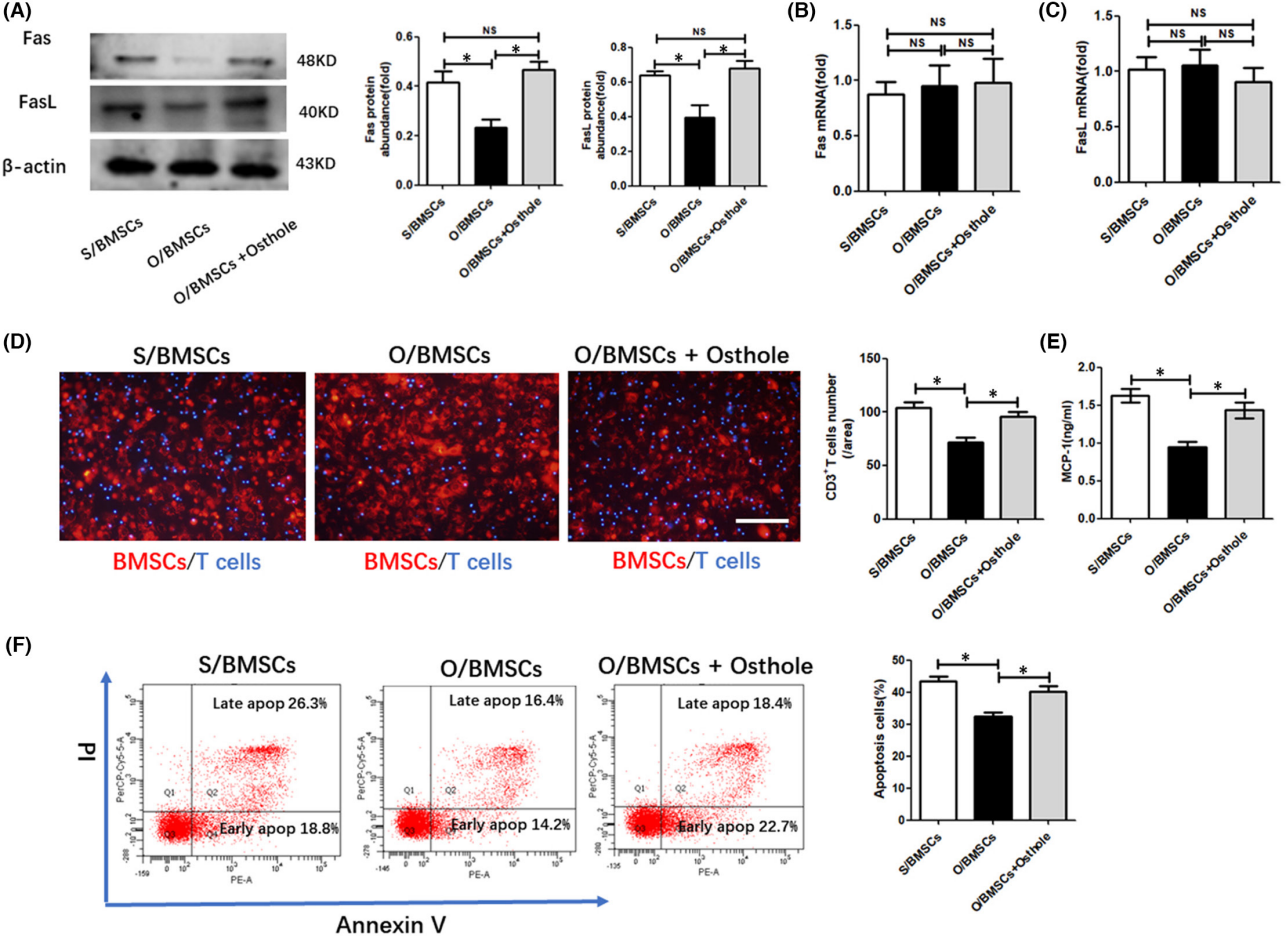
Osteoporotic BMSC‐induced T‐cell migration and apoptosis were rescued by osthole. (A) Western blot of Fas and FasL protein accumulation in BMSCs. ImageJ was used to measure the relative protein abundance. (B,C) The mRNA levels of Fas and FasL were measured with real‐time PCR. (D) T cells were cocultured with S/BMSCs, O/BMSCs and O/BMSCs pre‐treated with osthole. A fluorescence microscope was used to quantify and observe T‐cell migration. Scale bar, 100 mm. (E) MCP‐1 levels in the culture medium of S/BMSCs, O/BMSCs and O/BMSCs pre‐treated with osthole were measured with ELISA. (F) Flow cytometry was used to analyse apoptotic T cells induced by S/BMSCs, O/BMSCs and O/BMSCs pre‐treated with osthole. NS, not significant; data presented as mean = ±SD. *p < 0.05.

**FIGURE 2:**
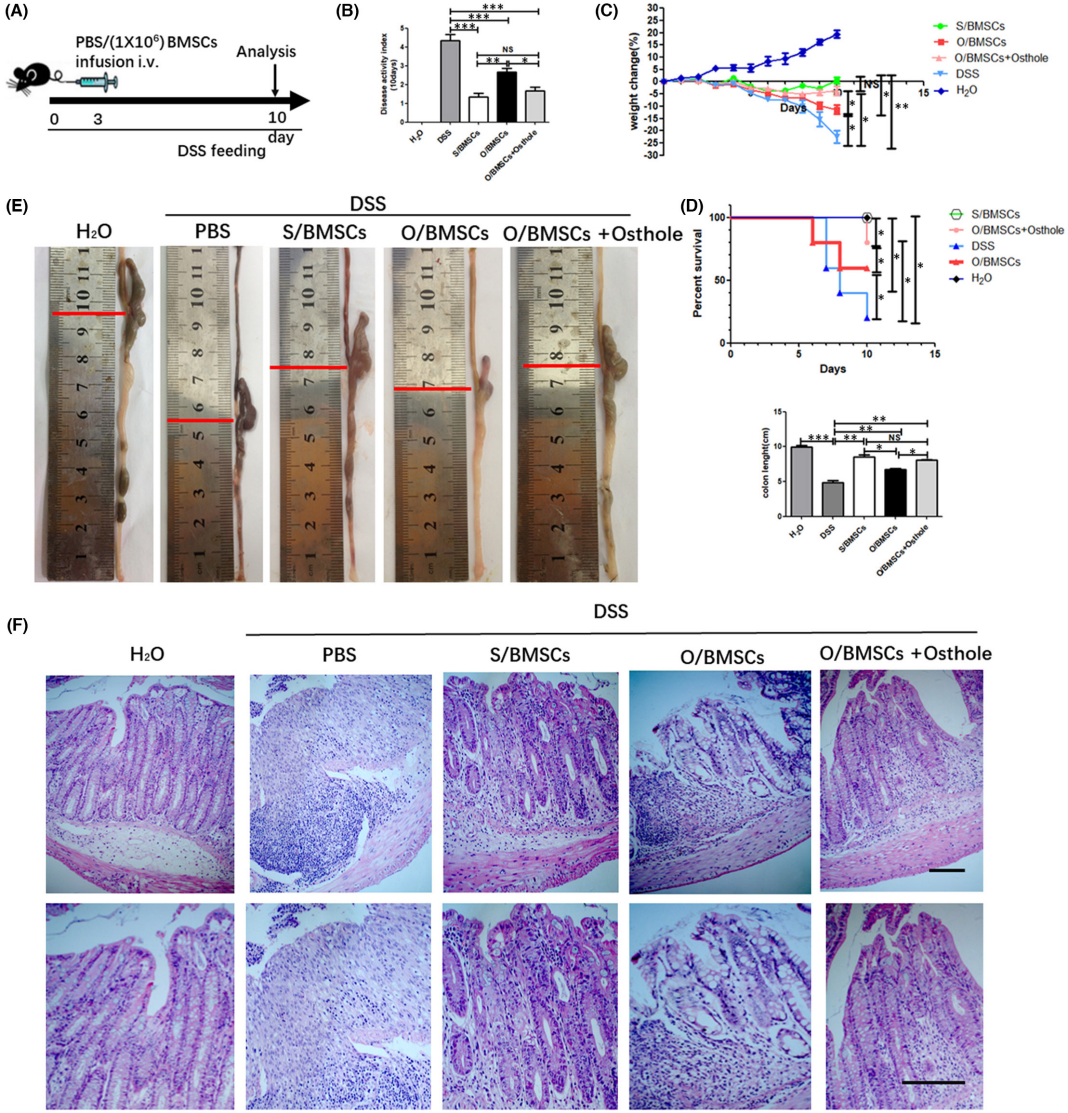
Treatment of inflammatory colitis with BMSCs. (A) Experimental design. Mice were fed drinking water containing DSS for 10 days. On the third day, 1 × 10^6^ S/BMSCs, O/BMSC and O/BMSCs pre‐treated with osthole were injected into the mice through the tail vein. (B) The disease index was measured on the 10th day of DSS feeding. (C) Bodyweight was recorded every day for 10 days. (D) The mortality of the mice was recorded at 10 days. (E) The colon was collected 10 days later for each group, and the length was measured. (F) The histological structure of the colon was determined using H & E staining, and the histological score was graded. The bottom image is a higher magnification of the top image. Scale bar, 200 mm. (G) Ratio of apoptotic CD3 + T cells. (H–K) ELISA analysis of serum levels of inflammation markers TNF‐α, IFN‐γ, IL‐1β and IL‐6. NS, not significant; data displayed as means ± SD. *N* = 5/group. *p < 0.05 and **p < 0.01.

**FIGURE 4:**
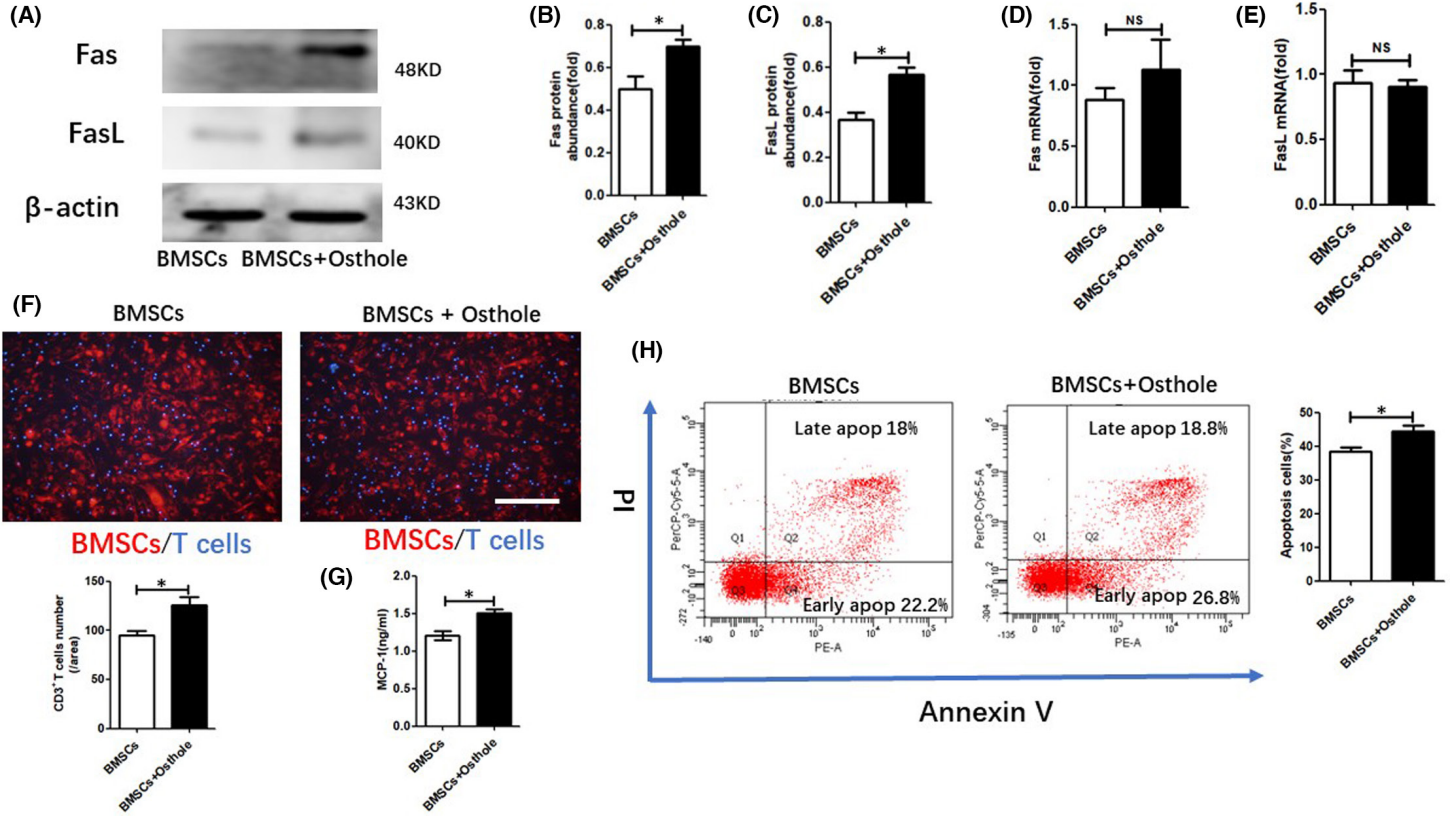
Osthole increased BMSC‐induced T‐cell migration and apoptosis. (A–C) Western blot was used to visualize the accumulation of Fas and FasL proteins in BMSCs. ImageJ software was used to measure the relative protein abundance. The grey value of each stain was normalized to the value of β‐Actin. (D,E) Real‐time PCR was used to measure the mRNA levels of Fas and FasL. (F) T cells were cocultured with BMSCs pre‐treated with osthole. Under a fluorescence microscope, T‐cell migration was counted and quantified. Scale bar, 100 mm. (G) MCP‐1 level in BMSC culture medium was measured with ELISA. (H) Flow cytometry analysis of apoptotic T cells. NS, not significant; data are shown as means ± SD. *p < 0.05

**FIGURE S1:**
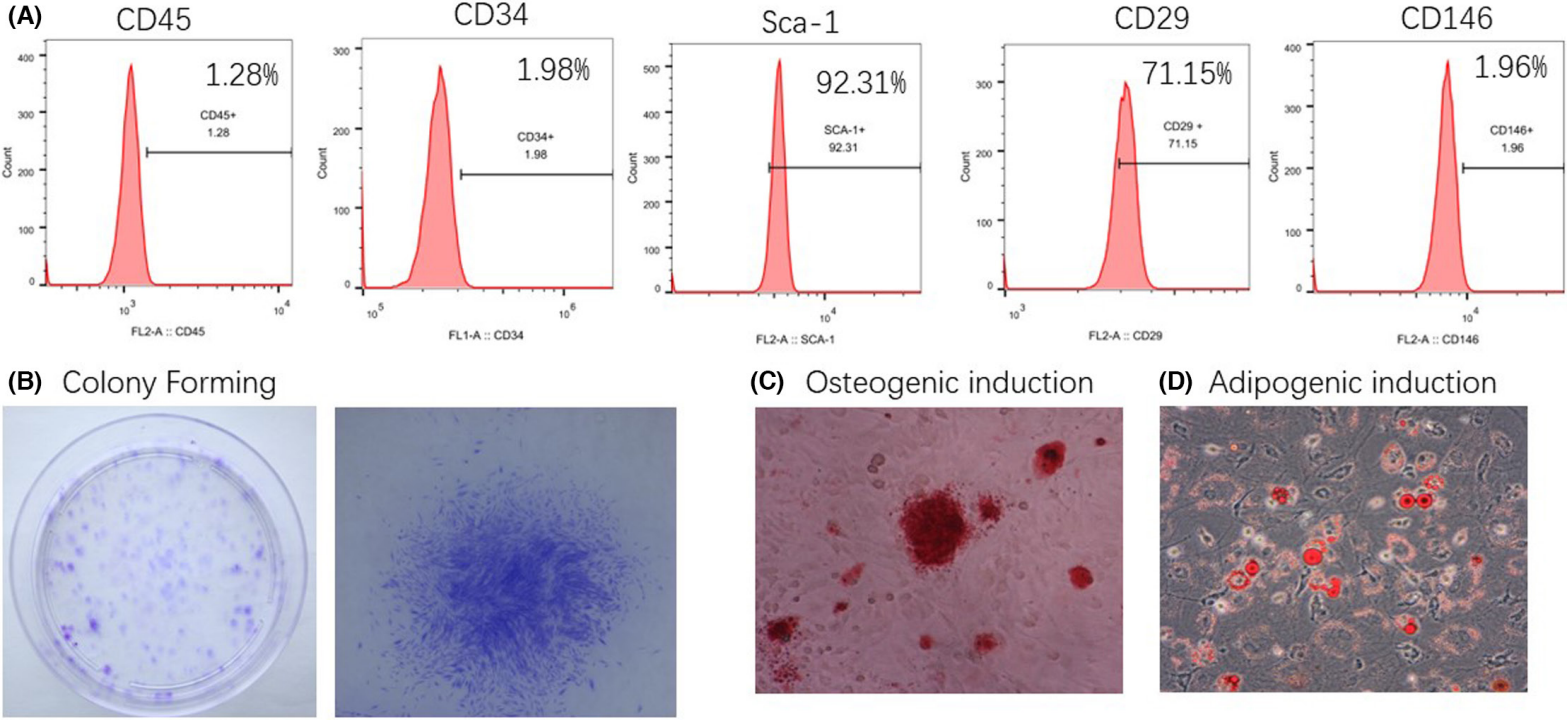
Identification of the characteristics of BMSCs. (A) Flow cytometry was used to determine the surface markers expressed in BMSCs. (B) Colonies formed by BMSCs were tested with methionine blue staining. (C) After 21 days of osteogenic induction, alizarin red staining was used to test the formation of mineralized nodules. (D) After 14 days of induction, Oil red O staining was used to test adipogenic differentiation.
