# Supplementary figures and images for: Osthole enhances the immunosuppressive effects of bone marrow‐derived mesenchymal stem cells by promoting the Fas/FasL system
Source: J Cell Mol Med. 2021 Mar 21;25(10):4835–45. doi: 10.1111/jcmm.16459 (PMC8107110; doi:10.1111/jcmm.16459)

**Figure S**


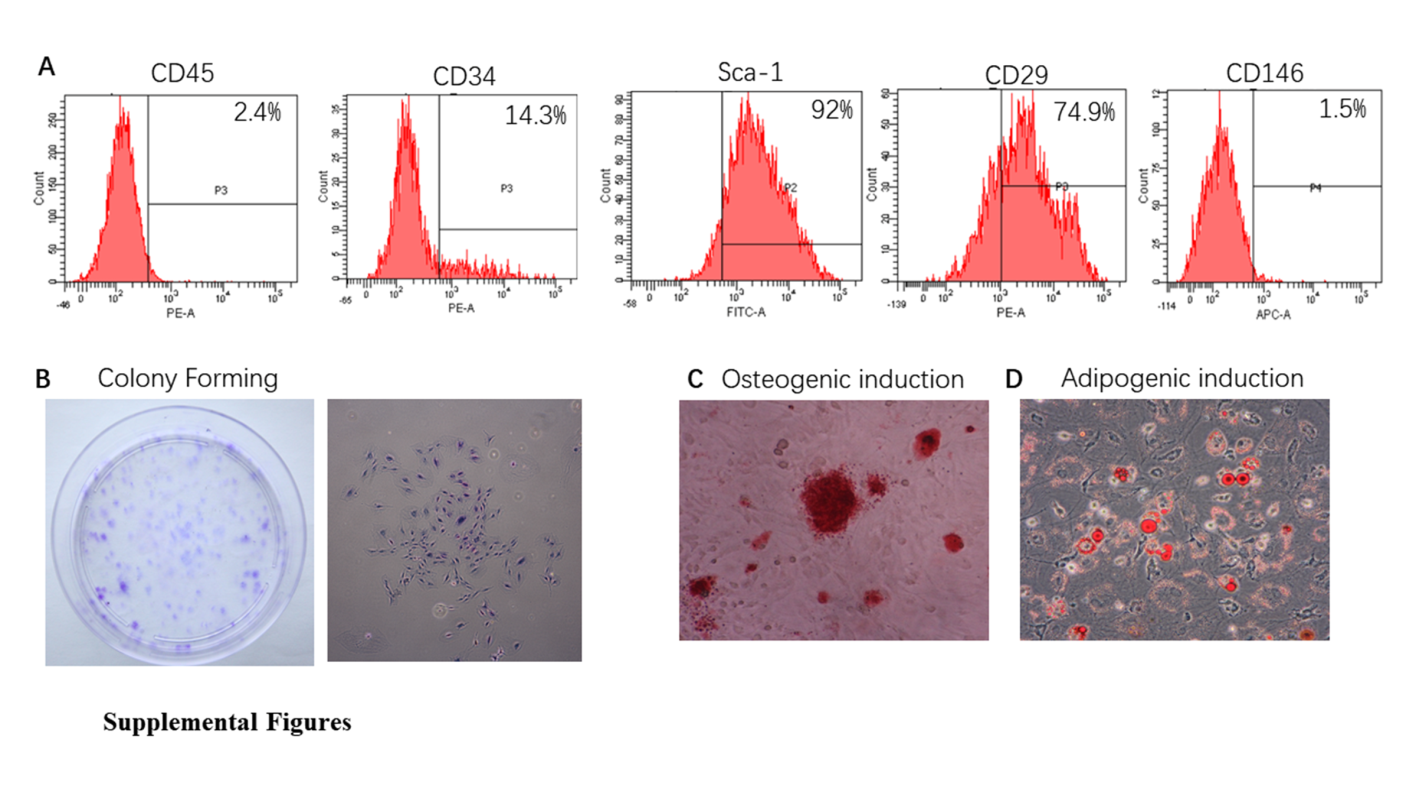

Supplement: Supplementary file 1 — Fig S1 [file JCMM-25-4835-s001.docx]
